# Screening genome-wide DNA methylation CpG sites via training and testing data utilizing surrogate variables

**DOI:** 10.1186/1471-2105-15-S10-P4

**Published:** 2014-09-29

**Authors:** Meredith Ray, Xin Tong, Hongmei Zhang, Wilfred Karmaus

**Affiliations:** 1 Department of Epidemiology and Biostatistics, University of South Carolina, Columbia, SC 29208, USA; 2 Division of Epidemiology, Biostatistics, and Environmental Health, University of Memphis, Memphis, TN 38152, USA

## Background

Screening Cytosine-phosphate-Guanine dinucleotide (CpG) DNA methylation sites in association with single-nucleotide polymorphisms (SNPs), or covariate of interest, and/or their interactions is desired before performing more complicated analyses due to high dimensionality. It is possible the variation in methylation cannot be fully explained by SNPs and covariates of interest and thus it is important to account for variations introduced by other unknown factors. Furthermore, CpG sites screened from one data set may be inconsistent with those from another data set and it is equally important to improve the reproducibility of the selected CpG sites.

## Materials and methods

A user-friendly R package, training-testing screening method (ttScreening), was developed to achieve these goals and provides users the flexibility of choosing different screening methods: proposed training and testing method, a method controlling false discovery rate (FDR), and a method controlling the significance level corrected by use of the Bonferroni method.

## Results

Linear regressions were applied in the screening process, with methylation of a CpG site as the dependent variable, a single SNP, a covariate, and possibly their interactions as independent variables. Surrogate variable analyses were included to adjust for unknown factor effects. Randomly chosen training and testing samples were used to estimate and test the effects, respectively. Simulations based on different scenarios were implemented to test the robustness and sensitivity of the method and to compare with the other two screening methods. For almost all simulation scenarios, the training and testing screening method proved to outperform other methods in terms of correct identification of important CpG sites. For other occasions, ttScreening performed equally well. We applied ttScreening to 40,000 CpG sites based on their association with smoking and forced vital capacity. The ttScreening method selected 9 CpG sites and the other two methods selected 0 CpG sites.

## Conclusions

Our simulation results indicate that ttScreening performs better than FDR-based screening and that it is at least as good as Bonferroni in terms of correctly identifying CpG sites that are associated with other variables. The package is computationally efficient and user-friendly, which indicates its suitability to high dimensional data for dimension reduction and its broad application in addition to epigenetic studies. The package can be downloaded at [1].
